# Agricultural water policy reforms in China: a representative look at Zhangye City, Gansu Province, China

**DOI:** 10.1007/s10661-017-6370-z

**Published:** 2017-12-07

**Authors:** Tomohiro Akiyama, Ali Kharrazi, Jia Li, Ram Avtar

**Affiliations:** 1 Graduate School of Frontier Sciences, The University of Tokyo, Tokyo, Japan; 2 Graduate School of Public Policy, The University of Tokyo, Tokyo, Japan; 3 Advanced Systems Analysis Program, International Institute for Applied Systems Analysis (IIASA), Laxenburg, Austria; 4 Faculty of International Studies and Regional Development, University of Niigata Prefecture, Niigata, Japan; 5 Graduate School of Environmental Science, Hokkaido University, Sapporo, Japan

**Keywords:** Agricultural water policies, Land use/land cover, Vegetation indices, Landsat

## Abstract

Water resources are essential for agricultural production in the grain-producing region of China, and water shortage could significantly affect the production and international trade of agricultural products. China is placing effort in new policies to effectively respond to changes in water resources due to changes in land use/land cover as well as climatic variations. This research investigates the changes in land, water, and the awareness of farmer vis-à-vis the implementation of water-saving policies in Zhangye City, an experimental site for pilot programs of water resources management in China. This research indicates that the water saved through water-saving programs and changes in cropping structure (2.2 × 10^8^ m^3^ a^−1^) is perhaps lower than the newly increased water withdrawal through corporate-led land reclamation (3.7 × 10^8^ m^3^ a^−1^). Most critically, the groundwater withdrawal has increased. In addition, our survey suggests that local government is facing a dilemma of water conservation and agricultural development. Therefore, the enforcement of the ban on farmland reclamation and irrigation water quotas in our study area is revealed to be relatively loose. In this vein, the engagement of local stakeholders in water governance is essential for the future sustainable management of water resources.

## Introduction

China’s rapid development and large population exert serious strains on its water resources. Water resources in China continue to face severe management constraints and critically challenge the country’s social, economic, and environmental sustainability (Yong 2009). Especially, growing demand for irrigation water and limited management practices are placing pressure on water resources (Liu et al. 2017). Previous water resource management strategies focusing on water conservancy and transfer projects, e.g., irrigation canals and dams, have allowed China to leverage its limited water resources and arable land to feed its large population. Although China’s annual average freshwater resources of 2800 billion cubic meters is ranked as the sixth largest in the world, the per capita water resources of the country stands at a mere 2040 m^3^ per capita which are only one fourth of the global average (Wang et al. 2008). In addition to the low per capita availability of water resources, there is a mismatch in China between the spatial distribution of water resources and geographic regions with high population densities.

Policy and decision makers in China continue to pay significant attention to the role of water resources in sustainable development. In this avenue, two management approaches have been advanced. From the early years of the establishment of the People’s Republic of China in 1949, the country’s water resource management approach has been dominated by the hard path approach (Gleick 2003). The hard approach emphasizes investments in water infrastructure, e.g., dams, irrigation canals, dredging, reservoirs, to both increase water supply and water consumption efficiency. More recently, since the 1990s, the country has witnessed efforts to transition from hard path to soft path approach emphasizing economic and institutional water resource management strategies (Chen et al. 2007; Gu et al. 2017). The soft path approach has focused on regulatory instruments, e.g., the 1991 Law on Water and Soil Conservation, 1996 Law on Prevention and Control of Water Pollution, 1997 Flood Control Law, and the 2002 Water Law; economic instruments, e.g., water pricing, tariffs, and quotas; and stakeholder capacity building, e.g., public education and knowledge diffusion (Liu et al. 2013). Despite the considerable achievements in the management of water resources in China, more efforts need to be directed towards removing the social and cultural obstacles, most notably, socio-cultural obstacles which prevent the successful implementation of soft path approach, e.g., the enforcement and public participation of water management policies and regulations (Liu and Yang 2012).

The challenges of water resource management are perhaps most severe in the Heihe River basin, an important grain-producing semi-arid and arid northern region of the country, where the exploitation of existing water resources has resulted in a growing water shortage problem. In the Heihe River basin, approximately 80% of the freshwater is consumed by irrigated agriculture (Chen et al. 2005; Yang et al. 2015) and therefore, previous hard and soft path water management approaches have focused on this important sector. In addition to common technical efforts, e.g., improvement of irrigation infrastructure efficiency, important institutional management efforts such as water pricing, cropping pattern optimization, and regulatory frameworks have also been introduced. However, it is unclear how effectively institutional management efforts and especially regulatory frameworks are being implemented. One of the biggest obstacles in this avenue is the public perception of water as a public rather than economic good. Through the increased knowledge capacity of stakeholders, e.g., farmers, agricultural companies, and local government officiers, these perceptions are being gradually changed and water use rights, regulations, and market-based management mechanisms are being clarified and better implemented.

It is still unclear whether policy changes induce water conservation while both meeting the growing water demands of agricultural production, municipal, and industrial sectors. Therefore, it is warranted to evaluate the effectiveness of agricultural water policy reforms and the role and awareness of local farming stakeholders of water governance challenges. Towards this end, this study adopts the use of geospatial data to monitor changes in land use and land cover. It is essential to link the pattern of land cover change to the underlying mechanisms and how the change in cropping pattern affects the water demand to adopt new agricultural water policies. Change in China’s agricultural water policies will affect the agricultural production and therefore it is essential that China’s water management and policy institutions encourage effective water conservation. This paper is organized as follows: “The study area: the Heihe River basin and Zhangye City” section introduces the study area. The “Materials and methods” section discusses the materials and methods employed in this paper. The “Results” section overviews the results from the spatial analysis, statistical data, and questionnaire survey. Finally, a conclusion and discussion of the research findings follow in the “Discussion and conclusion” section.

## The study area: the Heihe River basin and Zhangye City

**Fig. 1:**
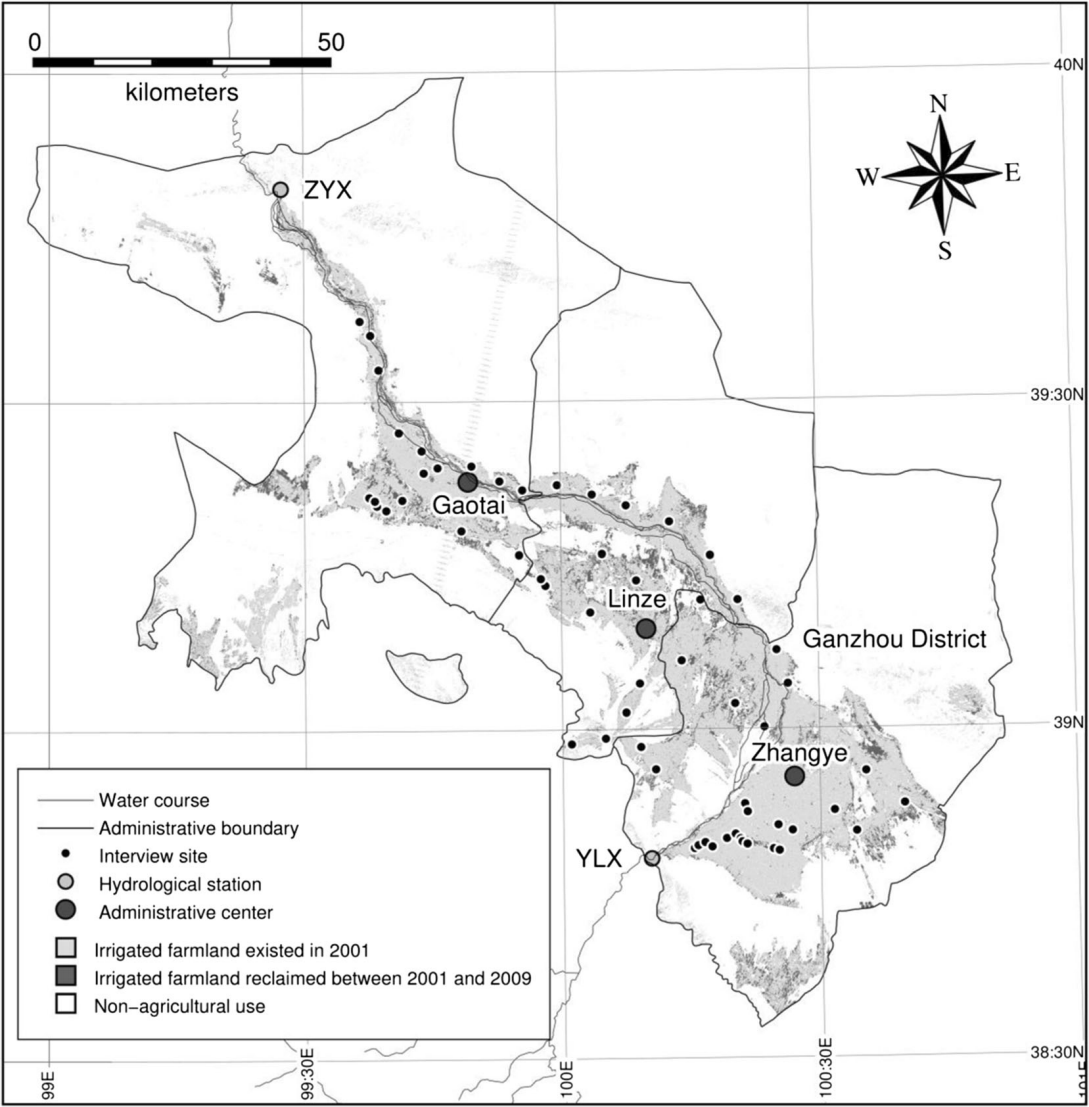
Map of the middle reach of the Heihe River basin and the survey sites. This study covers one municipal district and two counties of Zhangye City, wherein the mainstream of the Heihe River flows through, i.e., Ganzhou District, Linze County and Gaotai County from the southeast to northwest. Light gray and dark gray respectively indicate irrigated area in 2001 and 2009 derived from remotely sensed data in three counties of Zhangye

The Heihe River feeds on melting glaciers and precipitation in the Qilian mountains and flows through three provinces in Northwestern China, i.e., Qinghai, Gansu, and Inner Mongolia. The Heihe River basin is the second largest inland river basin in China, which can be divided into three reaches with boundaries at sites Yingluoxia (YLX) and Zhengyixia (ZYX) hydrological stations (Fig. 1): the upper mountainous, middle oasis, and lower desert reaches. The upper reaches are cold mountainous areas where glaciers have significantly shrunk, approximately by 138.90 km^2^, during the period from 1960 to 2010 (Qiang et al. 2014). The middle reaches consist of alluvial fans with a cultivated area of 1696 km^2^ in 2009. The Heihe River Basin provides the majority of water resources for agricultural development. However, low agriculture water use efficiency has led to a severe mismatch between water supply and demand (Liu et al. 2017). The irrigation period in this region is from April to September. The lower reaches are an alluvial and lacustrine plain underlaid with unconsolidated sediments from the Quaternary age. The lower reaches include a large expanse of desert land and sparse vegetation. The annual ranges of precipitation in the upper, middle, and lower reaches are, respectively, 300 to 500 mm, 100 to 300 mm, and less than 100 mm. More than 90% of the precipitation in all the reaches occurs from April to September. Among the three reaches, abundant and available water resources, i.e., both surface water and groundwater, are mainly found in the middle reaches. Given the availability and abundance of water, irrigation and agricultural development have been significantly increased in the middle reaches since the 1950s.

This research focuses on Zhangye City in the Gansu Province which is located in the middle reaches of the Heihe River basin. Zhangye is the largest oasis in the Heihe River basin and where the vast majority of the farmland of the river basin is located. In Zhangye City, the surface water and groundwater account for approximate 67% and 30% of the total water consumption of the river basin (Liu et al. 2017). The jurisdiction of Zhangye City includes Ganzhou District, Linze County, Gaotai County, Sunan County, Shandan County, and Minle County. The mainstream of the Heihe River flows through Ganzhou, Linze, and Gaotai.

Zhangye City’s economy is heavily dependent on agricultural production. Due to extensive irrigation farming development since the 1950s, the environment in the lower reaches has gradually degraded. In the late 1990s, Zhangye City came under the national spotlight of water disputes between agricultural irrigation in the middle reaches against environmental recovery in the lower reaches. Especially, the decrease in river discharge to the lower reaches and decline of groundwater level caused the two terminal lakes, the West Juyan lake with an area of 267 km^2^ and the East Juyan lake with an area of 35 km^2^, to become dry respectively in 1964 and 1991 (Wang and Cheng 1999). The drying lakes were considered as one of the reasons for sandstorms in northern China and have attracted attention from both mass media and policymakers.

Given the national focus, the Chinese government since 2000 has initiated two comprehensive programs in the Heihe River basin. Zhangye City, consequently, became a “laboratory” for new policies, such as the Water Users’ Association (WUA) policies and water quotas for irrigation. These policies led to two programs which played the role of a showcase for the water governance regime of the country. The first program was the short- to mid-term governance program of the Heihe River Basin. This program, initiated in 2001, aimed to enforce an integrated water resource management policy in the river basin by dividing water availability among regions based on quantitative targets (Ministry of Water Resources 2001). The second program was The Construction of a Water-Saving Society. This program, initiated in 2002, aimed to establish a participatory water management system with clearly defined water use rights (WUR) and tradable water quotas. This program was also aimed at releasing more water to the lower reaches by conserving irrigation water. This included the restriction of river water usage, encouragement of groundwater use, restrictions on expanding farmland, and the introduction of crops, i.e., maize, which consume less water. The final completion of both programs was scheduled for the year 2010.

## Materials and methods

This study adopts three analytical methods. Firstly, we conducted spatial analysis to examine land use and land cover change (LUCC) in Zhangye City in the Ganzhou District between 2001 and 2009 using remotely sensed data. Secondly, we collected statistical data of water withdrawals while we estimated water consumption of the study area by calculating crop evapotranspiration from irrigated farmlands. Thirdly, we conducted a semi-structured survey questionnaire to investigate the awareness of the local farmers and their recognition of the new water management policies.

### Estimation of land use and land cover change

We made a classification of land use and land cover in the middle reaches of the Heihe River basin to estimate evapotranspiration from irrigated farmlands—perhaps one of the most important components of the water balance analysis. Towards this end, we focused on the years 2001 and 2009 to identify LUCC before and after the implementation of the water-saving policy. To perform the classification, the study proceeded in five steps: (i) data collection of remotely sensed data as well as ancillary data, (ii) pre-processing of the remotely sensed data, (iii) supervised classification of land use and land cover for the year 2009, and (iv) the development of a decision tree algorithm. Upon completion of all these processes, we evaluated the accuracy of the classifications. The GRASS GIS (version 6.4.3) software was used for the classifications and data analyses. Land use plays important role in water resources allocation and influence regional water resource utilization. Water and land resources have been closely linked together in integrated river basin management. Thus, it is of great significance to take land use as a critical factor in water allocation in river basins. This echoes the work of Wang et al. (2015) in considering land use as a significant constraint of the optimal water resources allocation plan.

#### Data collection

Table 1 summarizes the remotely sensed data used in the study. We chose Landsat TM/ETM+ data with a spatial resolution of 30 m which is sufficient to identify crop types in single agricultural fields which are generally small in the study area. Considering the growing season of various crops in the study area, we selected the Landsat TM/ETM+ data acquired in April, June, August, and October with minimum cloud cover. The data was obtained from the US Geological Survey’s (USGS) Earth Resources Observation and Science Center (EROS) and the China Remote Sensing Satellite Ground Station.

**Table 1:** Remotely sensed data used in the analysis of land use/cover change

Satellite	Sensor	Bands	Resolution	Acquisition date	Path	Row	Data source
Landsat 5	TM	7	30 × 30 m	15-Apr-2001	133	33	USGS
				18-Jun-2001	133	33	USGS
				21-Aug-2001	133	33	USGS
				12-Aug-2001	134	32	USGS
				28-Aug-2001	134	32	USGS
				24-Oct-2001	133	33	USGS
				21-Apr-2009	133	33	CAS
				12-Apr-2009	134	32	CAS
				12-Apr-2009	134	33	CAS
				24-Jun-2009	133	33	USGS
				1-Jul-2009	134	32	USGS
				1-Jul-2009	134	33	USGS
				11-Aug-2009	133	33	USGS
				17-Jul-2009	134	32	USGS
				17-Jul-2009	134	33	USGS
				14-Oct-2009	133	33	CAS
				21-Oct-2009	134	32	CAS
				21-Oct-2009	134	33	CAS
Landsat 7	ETM+	7	30 × 30 m	30-Apr-2001	134	32	USGS
				30-Apr-2001	134	33	USGS
				17-Jun-2001	134	32	USGS
				17-Jun-2001	134	33	USGS
				20-Aug-2001	134	33	USGS
				23-Oct-2001	134	32	USGS
				23-Oct-2001	134	33	USGS

For the development of the decision tree algorithm, we conducted interviews with the local water authorities, farmers in the study area, and collected crop calendar data. Crop calendar data includes information on the types of irrigated crops, planting and harvesting dates, and periods of plant growth. We also used the leaf area index data to interpret seasonal patterns of crops. The leaf area index is the ratio of total upper leaf surface of vegetation divided by the surface area of the land on which the vegetation grows. It is a dimensionless value, typically ranging from 0 for bare grounds to 6 for a dense forest.

Ground-truth data was collected for the purpose of supervised classification and classification accuracy assessment. We collected ground truth data with the help of local farmers on different vegetation types in different areas of the middle reaches of the basin in January 2010. The ground-truth data includes seven categories of land use and land cover in the study area and in total 307 sites. Furthermore, we collected statistical data from survey departments to investigate historical changes in irrigated farmland areas by crop type.

#### Pre-processing

LUCC detection requires accurate geometric and radiometric correction of the data to enable a meaningful analysis, necessitating a rigorous pre-processing scheme for all the Landsat TM/ETM+ data. Towards this end, we first performed geometric corrections, using ground control points (GCPs) as provided by USGS-EROS, to make the image features geographically correct. The individual Landsat scenes were geo-referenced in the UTM projection based on WGS84 datum. Secondly, we mosaicked the data in order to achieve a full coverage for the region of interest, i.e., the middle reaches of the Heihe River basin. Thirdly, we performed radiometric corrections to convert digital numbers into spectral reflectance using sensor calibration parameters (Avtar et al. 2011; Chander et al. 2009).

Three spectral indices were calculated to identify cropland types: Normalized Difference Soil Index (NDSI), Normalized Difference Water Index (NDWI), and Optimized Soil-Adjusted Vegetation Index (OSAVI). The equations of NDSI, NDWI, and OSAVI are as follows (Pan et al. 2010):

1 $$ \mathrm{NDSI}=\frac{\rho_{\mathrm{band}5}-{\rho}_{\mathrm{band}4}}{\rho_{\mathrm{band}5}+{\rho}_{\mathrm{band}4}} $$

2 $$ \mathrm{NDWI}=\frac{\rho_{\mathrm{band}3}-{\rho}_{\mathrm{band}5}}{\rho_{\mathrm{band}3}+{\rho}_{\mathrm{band}5}} $$

3 $$ \mathrm{OSAVI}=\frac{\rho_{\mathrm{band}4}-{\rho}_{\mathrm{band}3}}{\rho_{\mathrm{band}4}+{\rho}_{\mathrm{band}3}+0.16} $$

where *ρ*_band3_, *ρ*_band4_, and *ρ*_band5_ are the spectral reflectances of Landsat TM/ETM+ bands 3, 4, and 5, respectively. We used OSAVI as suggested by Rondeaux et al. (1996).

#### Supervised classification for the year 2009

Supervised classification is commonly used for LUCC analysis. It categorizes all pixels in the raster image to represent land use and land cover classes. We passed through three steps in order to obtain land use and land cover maps of the study area in 2009: (i) A total of 236 training sampling locations were chosen to encompass a full variety of potential classes of land use and land cover across the entire study area, (ii) pixel signature analysis provides statistical information about the expected classes regarding the pixel’s OSAVI, and (iii) the supervised classification to the time-series images of OSAVI was implemented using the maximum likelihood algorithm with pixel training dataset. The maximum likelihood classifier was employed since it is considered as one of the commonly used methods in supervised classification (Avtar et al. 2013).

#### Development of the decision tree algorithm and decision tree classification

**Fig. 2:**
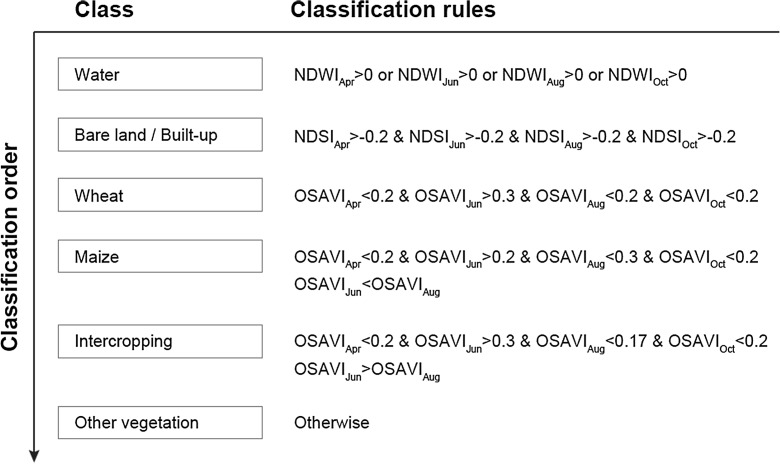
Rule base for decision tree classification at the object level

We developed a decision tree algorithm in order to classify land use and land cover in 2001. To do so, we used NDWI, NDSI, and OSAVI spectral indices in April, June, August, and October to identify water bodies and built-up areas as well as characterize the phenological patterns of crops. In order to classify farmland by crop, we derived classification rules based on time series data from the OSAVI index at a total of 71 training sampling locations as well as the cropping calendar data, the leaf area index data (Yamazaki et al. 2007), and the crop coefficient data. For example, classification rules for wheat and maize were established based on the differences in their growing seasons. More specifically, wheat is sown, sprouted, and harvested respectively around March 20, May 20, and July 12, while maize is sown, sprouted, and harvested respectively around April 16, July 20, and September 20. Furthermore, specific OSAVI index thresholds were parameterized by positive correlations between LAI and OSAVI. Figure 2 illustrates the proposed decision tree. It depicts how the land cover classes were individually differentiated and which index or assumptions were used at each step. For instance, water bodies were identified if the OSAVI index in either month was greater than 0 while bare land/built-up areas were identified if the NDSI index of every month was greater than − 0.06. The classification was done in a sequential way. Land use and land cover classification of the year 2001 was done using the newly developed decision tree algorithm. Classification accuracy analysis was performed against existing census data.

### Estimation of water withdrawal by source and by land category in 2001 and 2009

The sources of the irrigated water were derived from the river water and groundwater in the study area. In this vein, we collected statistical data of both river water withdrawal and groundwater withdrawal in 2001 and 2009 in the study area. To evaluate policy effectiveness, we further distinguished water withdrawals for the irrigated farmlands existing before the implementation of water-saving policies and the irrigated farmlands reclaimed after the implementation of water-saving policies. Since water withdrawal by farmland is not available, we made the assumption that water withdrawal is proportional to water consumption, that is, evapotranspiration from irrigated farmlands are as follows.

4 $$ \frac{RW_j+{GW}_j}{RW_i+{GW}_i}=\frac{ET_j}{ET_i} $$

where *RW* and *GW* denote the withdrawals of river water and groundwater, respectively (m^3^ year^−1^), *ET* denotes the crop evapotranspiration (m^3^ year^−1^), and suffixes *i* and *j* denote the year. Our field survey suggests that (Eq. 4) is reflective of the reality as local irrigation methods did not change significantly between 2001 and 2009.Specifically, flood or fallow irrigation were dominant while a negligible number of drip irrigation approaches were adopted in the study area during this period. Furthermore, the volume of irrigation frequency also did not significantly change during this period. Finally, the number of irrigation wells did not change significantly in the area where irrigated farmlands existed before the implementation of water-saving policies (refer to the light gray area in Fig. 1). Therefore, it is reasonable to assume an equal value of GW in both 2009 and 2001. This enables us to estimate the amount of river and groundwater withdrawal in 2009 for irrigated farmland reclaimed after the implementation of water-saving policies.

The total evapotranspiration of irrigated areas by crop type is computed by multiplying the reference evapotranspiration by the crop coefficient and total irrigated area. The equation used for the calculation is as follows:

5 $$ ET={K}_c\bullet {ET}_0\bullet A $$

where *ET* denotes the crop evapotranspiration (m^3^ year^−1^), *ET*_0_ denotes the reference evapotranspiration of irrigated area (mm year^−1^), *K*_*c*_ denotes the crop coefficient, and *A* denotes the irrigated area by crop (m^2^). The reference evapotranspiration coefficient is estimated following the method proposed by Kondo and Xu (1997). Note that this study assumes that the cloud cover is zero. The standard evapotranspiration is then estimated based on the heat balance of the surface using the daily mean air temperature, relative humidity, and wind speed observed at Zhangye meteorological station, which are derived from NOAA Global Surface Summary of the Day. The crop coefficients of the farmland and the landscape were respectively based on the research project “Historical evolution of adaptability in an oasis region to water resource changes” organized by Research Institute of Humanity and Nature^1^ and Liu et al. (2010).The irrigated areas by crop were based on the LUCC analysis presented in the “Pre-processing” section.

### Semi-structured questionnaire survey

**Fig. 3:**
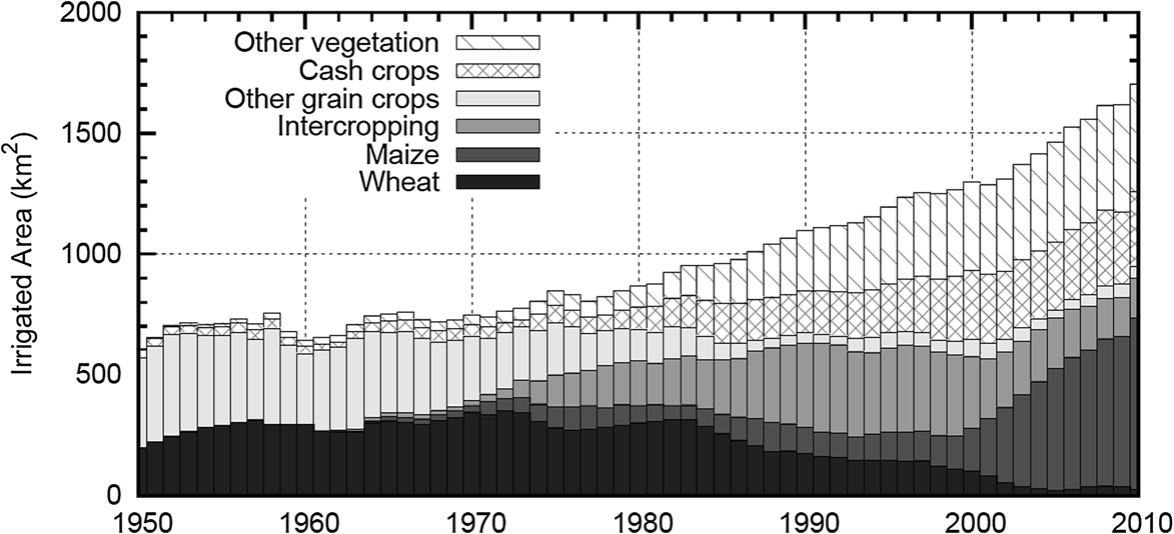
Changes in cropping structure derived from statistics

To investigate the awareness of the local farmers and their recognition of the new water management system, we conducted semi-structured questionnaire surveys during the period from February 23 to March 21, 2011. Respondents were asked about changes in their cropping structure (Fig. 3), irrigation volume and frequency, adoption of water-saving irrigation technologies, water management mechanisms, and agricultural market conditions. To generate a sample representative of Zhangye City, we mapped the study area through a 10-km grid dimension and visited the adjacent households on the corner points of the map. In total, we had 61 valid samples from 16 irrigation districts.

## Results

### Land use and land cover change

Land use/land cover classification is an important application of geospatial data which extracts thematic information from satellite data (Avtar et al. 2013). It is based on the spectral signature of various classes. In the study area, seven types of land use and land cover were classified, including maize, wheat, intercropping, other crops, other vegetation, water body, and bare land/built-up areas. Table 2 illustrates the farmland area by crop type derived from statistics, supervised classification, and the decision tree classification. Using the maximum likelihood supervised classification and decision tree classification (Table 2 (a)), the LULC map of the area reveals that most of the study area was covered by maize crops in 2009. In comparison to 2009, in 2001, most of the area was covered by intercropping (Table 2 (b)). In Table 2, a check was also performed against the existing census data. While previous studies have argued that Chinese official agricultural census data underestimates their cropland areas by 50% (Crook 1993; Fischer et al. 1998; Seto et al. 2000; Smil 1999; Xiao et al. 2002), agricultural census data can still be used to determine the fraction of crop area in each county (Frolking et al. 2002). In addition, the classification accuracy analysis was performed using ground-truth data to determine agreement between the selected reference pixels and the classified map. Table 3 illustrates accuracy assessments of the supervised classifications. The error matrix is used to describe the measure of accuracy between the images that have been classified and the training side of the same image. This approach is widely used and comprises the core of the accuracy assessment literature (Foody 2002). Accuracy assessments were carried out for each of the classification methods mentioned above, such as the overall accuracy, error matrix, and the Kappa coefficient. The overall accuracy of the classification was 90% with a 0.88 Kappa coefficient for images from 2009 and an overall accuracy of 69% with a 0.64 Kappa coefficient for images from 2001 (Table 3). This indicates a higher agreement between the ground and the satellite-derived map for 2009 in comparison to 2001.

**Table 2:** Irrigated farmland area by crop type derived from statistics, supervised classification, and decision tree classification: (a) Data for the year 2009 and (b) data for the year 2001

	Statistics				Supervised classification				Decision tree classification			
	Ganzhou District	Linze County	Gaotai county	Sum	Ganzhou District	Linze County	Gaotai county	Sum	Ganzhou District	Linze County	Gaotai county	Sum
(a)												
Maize	373.4	171.1	74.5	618.9	600.6	271.9	237.7	1110.2	638.5	240.6	208.7	1087.8
Wheat	34.4	0.7	3.3	38.4	34.4	2.6	0.1	37.1	54.4	8.2	1.4	64.0
Intercropping	95.6	20.3	45.7	161.6	210.8	66.2	29.7	306.7	132.9	79.2	45	257.1
Other crops	122.2	70.9	107.4	300.5	285.8	154.5	231.7	672.0	192.1	114.5	212.l	518.7
Other vegetation	151.4	132.6	157.5	441.5	143.9	112.2	168.3	424.5	229.5	139.4	156.4	525.3
Sum	777.0	395.5	388.5	1561.0	1275.5	607.4	667.5	2550.4	1247.4	581.8	623.S	2452.7
(b)												
Maize	145.5	71.2	20.4	237.1	N/A	N/A	N/A	N/A	251.2	72.7	66.5	390.3
Wheat	63.8	2.4	16.3	82.4	N/A	N/A	N/A	N/A	68.6	17.9	31.2	117.7
Intercropping	111.9	74.9	60.0	246.7	N/A	N/A	N/A	N/A	298.6	140.0	110.3	548.9
Other crops	115.6	67.1	101.6	284.3	N/A	N/A	N/A	N/A	193.5	84.6	129.0	407.0
Other vegetation	124.7	119.8	129.5	374.0	N/A	N/A	N/A	N/A	288.1	161.4	151.0	600.5
Sum	561.5	335.2	327.8	1225.0	N/A	N/A	N/A	N/A	1100.0	476.6	487.9	2064.0

*N/A* not available

**Table 3:** Accuracy assessment of land use and land cover classification at object level: confusion matrix, user’s and producer’s accuracy and overall accuracy: (a) the year 2009 and (b) the year 2001

	Maize	Wheat	Intercropping	Other crops	Other vegetation	Water	Bare land/built-up	Sum	User’s accuracy
(a)									
Maize	35	0	2	4	0	0	0	41	0.854
Wheat	0	17	0	0	0	0	0	17	1.000
Intercropping	2	0	14	1	0	0	0	17	0.824
Other crops	2	0	0	18	3	1	2	26	0.692
Other vegetation	0	0	0	3	32	0	0	35	0.914
Water	0	0	0	0	0	42	0	42	1.000
Bare land/built-up	0	0	0	1	0	2	55	58	0.948
Sum	39	17	16	27	35	45	57	236	
									Overall:
Producer’s accuracy	0.897	1.000	0.875	0.667	0.914	0.933	0.965		0.903
(b)									
Maize	34	0	5	3	2	0	0	44	0.773
Wheat	0	16	0	5	0	0	0	21	0.762
Intercropping	0	0	8	15	7	0	0	30	0.267
Other crops	4	0	0	2	6	0	9	21	0.095
Other vegetation	1	0	3	1	15	0	0	20	0.750
Water	0	1	0	0	1	44	4	50	0.880
Bare land/built-up	0	0	0	0	4	1	44	49	0.898
Sum	39	17	16	26	35	45	57	235	
									Overall:
Producer’s accuracy	0.872	0.941	0.500	0.077	0.429	0.978	0.772		0.694

### Change in distribution of irrigated farmland

**Fig. 4:**
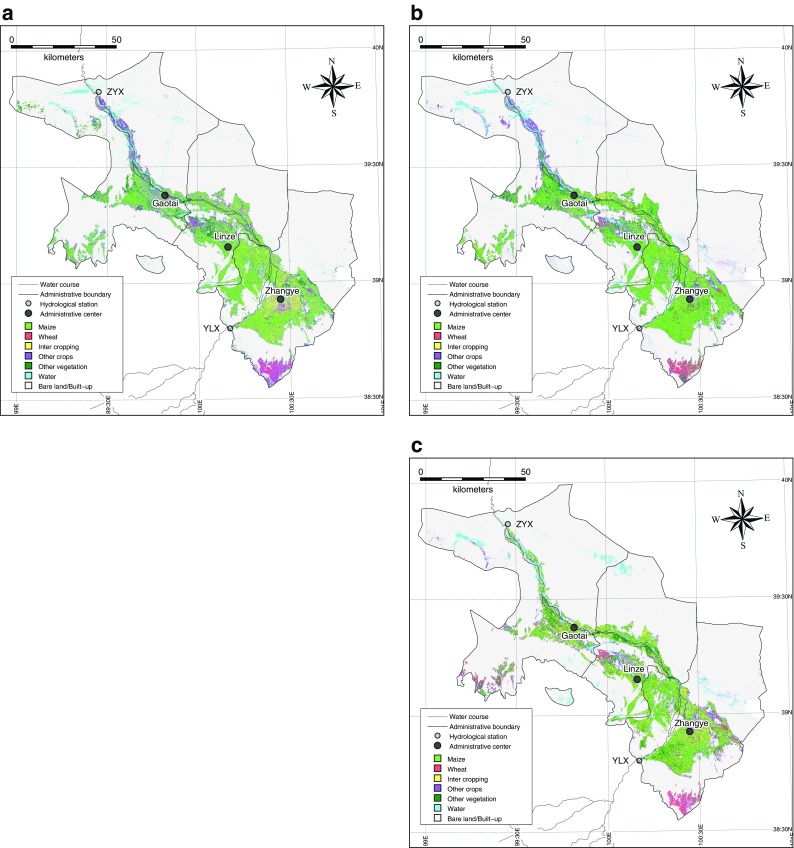
Map of the irrigated area. **a** Supervised classification for the year 2009. **b** Decision tree classification for the year 2009. **c** Decision tree classification for the year 2001

Figure 4 illustrates the changes in cropping structure derived from statistics. The cropping structure did change during the past 10 years. The crops for which cultivated area declined are wheat and intercropping. Substantial increases were observed for the maize crop. Since the 1980s, intercropping had become popular in Zhangye gradually due to its relatively high profit compared to the cultivation of single crops such as maize or wheat. In the 2000s, seed corn started to gain popularity among local farmer because. This was because first, given the contractual business model of the seed companies, there was a higher potential for achieving stable profits for farmers. Second, in comparison to other cash crops, e.g., vegetables and flowers, seed corn is less labor intensive. Therefore, it provides higher mobility to the rural population to work as migrant workers in more profitable non-farming urban sectors. It is noteworthy to mention that seed corn requires less irrigated water in comparison to intercropping, so various levels of local government also supported the diffusion of this crop among farmers.

Figures 1 and 4 show maps of the irrigated area in 2001 and 2009 derived from remotely sensed data in three counties of Zhangye City. The mainstream of the Heihe River flows through these three counties. In Fig. 1, the light gray colored areas indicate irrigated farmland which already existed in 2001, while the dark gray colored areas indicate new farmland which was reclaimed between 2001 and 2009. It is evident that land reclamation has continued in the fringe areas of the oasis even after the implementation of water-saving policies. Moreover, the ground-truth survey revealed that many of the new land reclamation reflected activities by agricultural corporations. In comparison to ordinary farmers, it is easier for corporations with new technologies and large financing capabilities to obtain permissions for digging wells and reclaiming farmland. Under the schemes of agricultural modernization and industrialization, these corporate activities were encouraged or at the very least not opposed by the government.

### Changes in water withdrawal by source and by land category

**Fig. 5:**
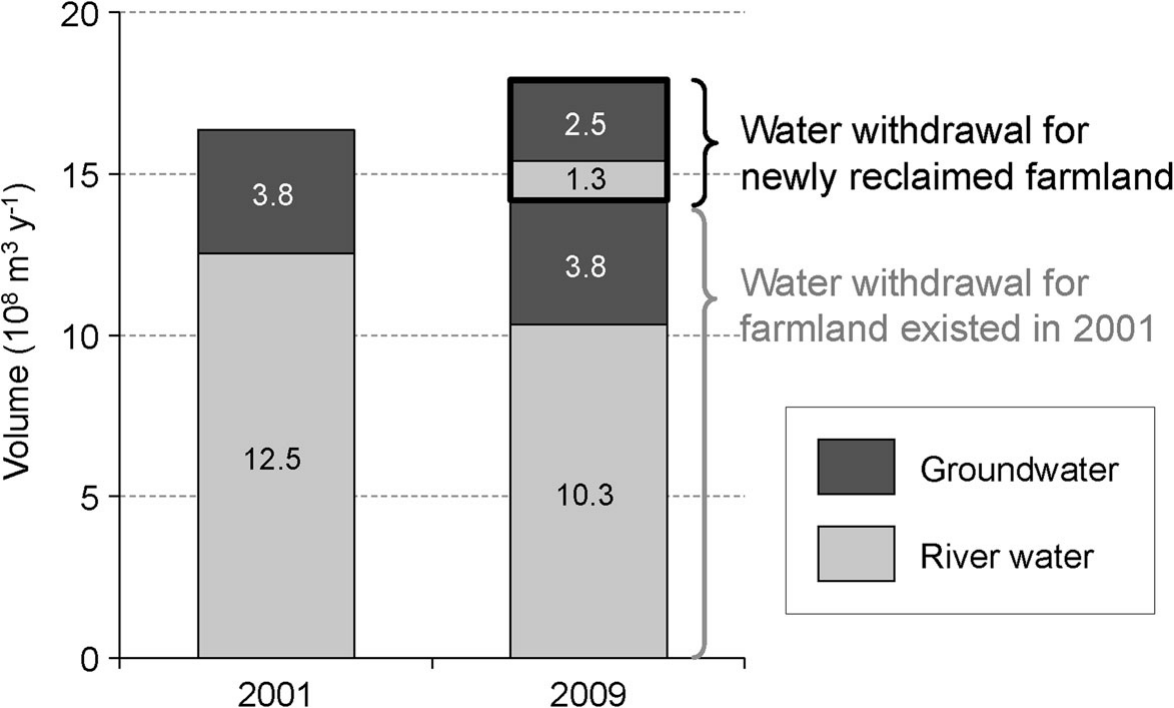
Changes in agricultural water consumption, based on estimated evapotranspiration, between 2002 and 2009

We estimated water withdrawals as well as water consumption, including evapotranspiration from irrigated farmland, respectively, in 2001 and 2009 by distinguishing the irrigated farmlands existing before the implementation of water-saving policies and the irrigated farmlands reclaimed after the implementation of water-saving policies. Figure 5 illustrates changes in estimated water withdrawals. Three important findings are revealed. Firstly, our results indicate that water-saving targets had been realized in 2009 in pre-existing farmlands. This is based on the observation that irrigated water sourced from the river to feed the same area of farmlands declined in 2001 from 12.5 × 10^8^ to 10.3 × 10^8^ m^3^ a^−1^ in 2009. Given the observation that less water was sourced from the river, the capacity of the pre-existing farmland areas in water conservation and efficiency had indeed been strengthened.

Secondly, our results indicate water withdrawal for pre-existing farmland areas declined from 16.4 × 10^8^ m^3^ a^−1^ in 2001 to 14.1 × 10^8^ m^3^ a^−1^ in 2009.However, newly reclaimed farmlands after 2001 required 3.7 × 10^8^ m^3^ a^−1^ more of irrigated water. This indicates that despite the successful implementation of water-saving policies in pre-existing farmland areas, the total agricultural water withdrawal, i.e., for pre-existing and newly reclaimed farmland areas, has in fact increased by 1.2 × 10^8^ m^3^ a^−1^ between 2001 and 2009. Furthermore, it also indicates that an increase of 3.7 × 10^8^ m^3^ a^−1^ water withdrawals, 2.5 × 10^8^ m^3^ a^−1^ of which comes from groundwater sources, for meeting the demand of newly reclaimed farmland areas. As the increase of water withdrawal relies mostly on groundwater sources, the threat to the groundwater capacity poses a new serious environmental threat and undermines the long-term resilience of water resources in the region.

Thirdly, our results indicate a fundamental challenge to equity and access to water resources. By examining closely the different stakeholders and beneficiaries of agricultural water, it seems that the implementation of water-saving policies has created an unjust balance in favor of agricultural corporations and at the expense of ordinary farming households. As illustrated by the dark gray colored areas of the map in Fig. 1, agricultural corporations have expanded their business activities in newly reclaimed farmland areas. To meet the water requirements, these corporations have mostly relied on groundwater sources. Therefore, from the water balance perspective, it is possible to argue that the agricultural corporations have exhausted the water conservancy achieved by ordinary farmers to irrigate their newly reclaimed farmland areas.

### Water quotas and actual water use in Ganzhou District

The two comprehensive governmental programs for water resource management in the region entailed the division of water based on quantitative targets, water use rights (WUR), tradable water quotas, and the introduction of new crops with lower water consumption rates. To examine the success of these policy tools, especially the WUR system and tradable water quotas, a survey of 25 households in the Ganzhou District, i.e., the most populated administrative area in Zhangye City, was conducted. We conducted the survey in Ganzhou because both the WUR and the water quota system were first introduced in this area and other counties followed these practices afterward. Through this survey, the differences between water quotas and actual water usage were examined. Table 4 illustrates the water quotas and actual water use in Ganzhou District for maize, wheat, intercropping, and vegetables for the summer/autumn and spring/winter irrigation periods. The results indicate higher actual water usage in terms of amount and frequency for most crops—the actual irrigation frequency for intercropping and vegetables met or were slightly below the quotas.

**Table 4:** Water quotas and actual water use in Ganzhou District. Notes: (1) We assumed that during summer/autumn irrigation, infiltration depth is 10 cm, while during spring/winter irrigation, infiltration depth is 15 cm. (2) In our sample, there are 26 households from Ganzhou District. Among them, 25 provided valid answers to our survey questions. (3) One mu equals to about 667 m^2^. (4) Vegetables grown in the households which we visited include bell pepper, chili pepper, tomato, Chinese cabbage, cauliflower, and potato. Data sources are Ganzhou District Government 2003 and the authors’ survey in 2011

		Maize	Wheat	Intercropping	Vegetables
Water quotas (m^3^/mu, per round)	Summer/autumn	75	75	75	65
	Spring/winter	85	85	85	85
Annual irrigation frequency quotas		5	4–5	7–8	10
Annual irrigation amount quotas (m^3^/mu, per round)		460	310–385	535–610	670
Actual irrigation amount (m^3^/mu, per round)	Summer/autumn	132	129	100	127
	Spring/winter	177	200	200	96
Actual annual irrigation frequency		6	6	8	9
Actual annual irrigation amount (m^3^/mu, per round)		811	769	900	828
Number of observations		22	2	1	7

Accordingly, it appears that the WUR and the tradable water quota system have not been firmly implemented in Zhangye City. These results can be partly explained through the change of the cropping structure after 2002 in Zhangye City from a high concentration of intercropping to a high concentration of seed corn. While seed corn requires a lower frequency of irrigation compared to intercropping (one or two fewer rounds), the actual water used for its cultivation continued to exceed the water quotas. Moreover, in addition to seed corn, the proportion of cash crops such as vegetables had also increased in the region. While vegetables require a lower volume of water in terms of each round of irrigation, their cultivation requires a higher frequency and therefore do not necessarily lead to any substantive savings in water consumption.

### Public participation in water management

Table 5 shows division of responsibilities in water management activities. Although many of the respondents knew about the WUA, they did not consider the new system as significantly different from the traditional system of collective management led by the village leaders. Farmers mentioned that the WUAs often composed of village leaders and production team leaders who are directly elected by the villagers. While the traditional system was not termed as a WUA, important issues regarding irrigation and farmland were equally discussed in local meetings where village leaders would facilitate a collective decision. Therefore, the WUA could be considered as an effort to institutionalize water management practices at the village level. When questioned who is responsible for various water management activities, respondents revealed that production team leaders and irrigation well managers are the key people to carry out all types of water management activities. In addition, only 11 of 61 respondents clearly knew who was responsible for purchasing and maintaining water tickets, which is the main instrument of tradable water use rights. In principle, water use rights are assigned to individual farming households whereby tickets are required to be purchased before the start of irrigation activities. However, in reality, production team leader often purchased water tickets from governmental authorities on behalf of all farming households in their team. Therefore, the majority of ordinary farmers would not receive a water ticket. This might be due to the top-down planning nature of irrigation schedules in the region, especially in the villages where river water consists of the main source of irrigation. Additionally, the farmland area for each household is too small to allow the trade of water use rights, which calls into question of the necessity to allocate water use rights to each individual farming household. Nevertheless, this top-down approach may have contributed towards the effective water trading practice between irrigation district consisting of multiple villages and industrial farming corporations.

**Table 5:** Division of responsibilities in water management activities. Notes: (1) Other responsibilities include, for example, production team meetings, water management offices, and working group. (2) In the case of well irrigation, electricity fees are also included in the water fee collections. (3) In foreground water/irrigation and well management, we inquired about the person who is keeping the key to the irrigation well(s)

	WUA		Village council		Production team leader/well manager		Ordinary farmers		Others		Total	
	Number of households	%	Number of households	%	Number of households	%	Number of households	%	Number of households	%	Number of households	%
Canal maintenance			12	16	48	66	7	10	6	8	73	100
Operation of sluice gates			7	10	31	44	26	37	6	9	70	100
Irrigation watchdog					9	24	26	70	2	5	37	100
Coordination of water delivery			5	9	29	53	1	2	20	36	55	100
Water fee collection			7	11	38	62			16	26	61	100
Groundwater/irrigation and well management			1	3	37	95			1	3	39	100
Purchasing/keeping water tickets			1	9	10	91					11	100

## Discussion and conclusion

Our study indicates that recent water policy reforms in Zhangye City may not meet the local government’s expectations. On one hand, the LUCC analysis indicates that, despite ecological restoration efforts in the 2000s, the total farmland area had in fact increased. On the other hand, the results from the analysis of the estimated water withdrawal and water consumption indicate that the newly reclaimed land requires (3.7 × 10^8^ m^3^ a^−1^) more water than the amount saved (2.2 × 10^8^ m^3^ a^−1^) through water policy reforms. Therefore, due to the expansion of farmlands after 2001, policies contributing to water conservation in Zhangye City were arguably not effective. In effect, to meet the demand of newly found farmland areas during this period, the pressure on river water sources had been replaced with a heavier dependence on groundwater sources. Furthermore, our survey indicates that more efforts need to be placed in familiarizing farmers in water-saving management practices and technologies. Farmers have only begun to adopt water-saving practices and the low levels of the adoption of these practices may be due to misalignment of incentives to the farmers.

However, these facts do not necessarily suggest the complete failure of water policy reforms. The changes in cropping structure did indeed induce the decline of irrigation volume for the same area of cultivated land. Furthermore, the level of public participation in water management is relatively high through self-governing institutions. The main players of water management, i.e., production team leader and well managers, are elected directly by the farmers. In some cases, management measures are formulated in the production team meetings to provide economic incentives and to maintain the local irrigation facilities.

The findings of this study suggest that, first, the local government is facing institutional constraints to save water as well as promote economic growth. This is perhaps best reflected through the tacit permission of the government towards farmland expansion. Second, reform policies are not necessarily superior to local practices. When designing water management reforms, China’s policymakers need to recognize that a one-size-fits-all policy approach does not exist. Reform of irrigation district management may be the key to the future success of water conservation policies in China. In this avenue, more effort needs to be placed in irrigation districts to establish a variety of management mechanisms and to promote water conservation among the stakeholders. The two most potentially successful reforms may be the establishment of more WUAs which are better engaged and able to incentivize the local stakeholders, especially farming households. Towards this end, it is essential for policymakers to examine the policy tradeoffs by considering direct feedback from various stakeholders to achieve the optimal allocation of water resources.

## Footnotes

1
http://www.chikyu.ac.jp/rihn_e/project/H-01.html
